# A clinical and molecular review of ubiquitous glucose-6-phosphatase deficiency caused by *G6PC3* mutations

**DOI:** 10.1186/1750-1172-8-84

**Published:** 2013-06-13

**Authors:** Siddharth Banka, William G Newman

**Affiliations:** 1 Manchester Centre for Genomic Medicine, Institute of Human Development, University of Manchester, Manchester, UK; 2 Manchester Centre for Genomic Medicine, Manchester Academic Health Science Centre, St. Mary’s Hospital, Central Manchester University Hospitals NHS Foundation Trust, Manchester, UK

**Keywords:** Ubiquitously expressed glucose-6-phosphatase enzyme, G-6-Pase, G-6-Pase 3, G6PC3, Severe congenital neutropenia type 4, Neutropenia, Dursun syndrome, Prominent superficial venous pattern, Congenital cardiac defects, Uro-genital anomalies

## Abstract

The *G6PC3* gene encodes the ubiquitously expressed glucose-6-phosphatase enzyme (G-6-Pase β or G-6-Pase 3 or G6PC3). Bi-allelic *G6PC3* mutations cause a multi-system autosomal recessive disorder of G6PC3 deficiency (also called severe congenital neutropenia type 4, MIM 612541). To date, at least 57 patients with G6PC3 deficiency have been described in the literature.

G6PC3 deficiency is characterized by severe congenital neutropenia, recurrent bacterial infections, intermittent thrombocytopenia in many patients, a prominent superficial venous pattern and a high incidence of congenital cardiac defects and uro-genital anomalies. The phenotypic spectrum of the condition is wide and includes rare manifestations such as maturation arrest of the myeloid lineage, a normocellular bone marrow, myelokathexis, lymphopaenia, thymic hypoplasia, inflammatory bowel disease, primary pulmonary hypertension, endocrine abnormalities, growth retardation, minor facial dysmorphism, skeletal and integument anomalies amongst others. Dursun syndrome is part of this extended spectrum. G6PC3 deficiency can also result in isolated non-syndromic severe neutropenia. *G6PC3* mutations in result in reduced enzyme activity, endoplasmic reticulum stress response, increased rates of apoptosis of affected cells and dysfunction of neutrophil activity.

In this review we demonstrate that loss of function in missense G6PC3 mutations likely results from decreased enzyme stability. The condition can be diagnosed by sequencing the *G6PC3* gene. A number of *G6PC3* founder mutations are known in various populations and a possible genotype-phenotype relationship also exists. G6PC3 deficiency should be considered as part of the differential diagnoses in any patient with unexplained congenital neutropenia.

Treatment with G-CSF leads to improvement in neutrophil numbers, prevents infections and improves quality of life. Mildly affected patients can be managed with prophylactic antibiotics. Untreated G6PC3 deficiency can be fatal. Echocardiogram, renal and pelvic ultrasound scans should be performed in all cases of suspected or confirmed G6PC3 deficiency. Routine assessment should include biochemical profile, growth profile and monitoring for development of varicose veins or venous ulcers.

## Review

## Introduction

**Figure 1:**
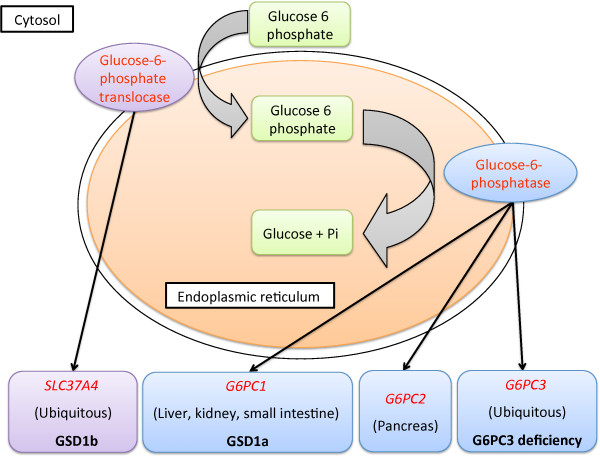
**The glucose-6-phosphatase system.** A schematic representation of the glucose-6-phosphatase system. Enzymes and genes are displayed in red font, expression sites of the genes are provided within brackets and associated disorders are highlighted in bold.

The glucose-6-phosphatase enzyme catalyzes the final step of glycogenolysis, hydrolysis of glucose-6-phosphate in the endoplasmic reticulum (ER). In humans, there are three glucose-6-phosphatase enzymes encoded by a gene-family consisting of *G6PC1*, *G6PC2* and *G6PC3*[1] (Figure 1). The enzymes encoded by the three genes, differ in their expression patterns and kinetic properties. *G6PC1* is expressed in the liver, kidney and small intestine. *G6PC2* is expressed mainly in the pancreas and *G6PC3* is ubiquitously expressed. The transport of glucose-6-phosphate from cytosol to ER is facilitated by glucose-6-phosphate translocase encoded by ubiquitously expressed *SLC37A4*.

Bi-allelic *G6PC1* mutations cause type 1a glycogen storage disease (GSD1a, MIM 232200) [2]. GSD1a is characterized by fasting hypoglycaemia, hepatomegaly, growth retardation, fasting lactic acidosis, hyperlipidaemia and hyperuricaemia. Long-term complications of GSD1a include hepatic adenomas, pancreatitis, gout, renal failure, pulmonary hypertension, polycystic ovaries, osteoporosis, platelet dysfunction, and mild to moderate learning difficulties [3]. Bi-allelic *SLC37A4* mutations result in glycogen storage disease 1b (GSD1b, MIM 232220) [4]. The phenotype of GSD1b is similar to that of GSD1a, but patients additionally develop chronic neutropenia.

The ubiquitously expressed human glucose-6-phosphatase enzyme (G-6-Pase β or G-6-Pase 3 or G6PC3, EC = 3.1.3.9), encoded by the *G6PC3* gene, was independently discovered by two groups in 2003 [5,6]. *g6pc3*^−/−^ mice demonstrate neutropenia, defects in neutrophil respiratory burst, chemotaxis, calcium flux, increased susceptibility to bacterial infection, elevated plasma glucagon and reduced plasma cholesterol [7,8]. In humans, homozygous or compound heterozygous *G6PC3* mutations cause an autosomal recessive multi-system syndrome of severe congenital neutropenia type 4 (SCN4 or G6PC3 deficiency, MIM 612541) [9]. We described the extended phenotype associated with the condition [10,11], and demonstrated that Dursun syndrome (MIM 613034) is part of this spectrum [12]. *G6PC3* mutations can also result in non-syndromic severe neutropenia [13,14].

In the last decade, significant progress has been made from the identification of the human ubiquitous glucose-6-phosphatase enzyme to understanding its role in physiology and disease. Here we provide an up-to-date review of the clinical, molecular and genetic aspects of G6PC3 deficiency.

### Epidemiology

Congenital neutropenia has an estimated minimum prevalence of 6 per million [15]. In addition to G6PC3 deficiency, mutations in *ELANE*, *GFI1*, *HAX1*, *CSF3R* and *WAS* are known to cause congenital neutropenia [16]. Neutropenia constitutes the phenotype of a number of multi-system conditions (listed under differential diagnoses). Xia et al. studied 162 patients from the North American severe congenital neutropenia tissue repository [17]. In this cohort, bi-allelic pathogenic *G6PC3* mutations were detected in two samples (1.2%), *ELANE* mutations in 55.6% of patients and no known genetic cause could be identified in about 40% of patients. In a recent British study of 108 kindreds that were negative for *ELANE*, *HAX1* and *WAS* mutations, four patients from three families were diagnosed with G6PC3 deficiency (2.8%) [14]. These two studies provide an estimate of the frequency of G6PC3 deficiency, but the true prevalence of the condition remains unknown. To date, at least 57 patients with G6PC3 deficiency have been described in the literature (Tables 1, 2 and 3).

**Table 1:** **Patients with homozygous missense *G6PC3 *mutation**

**Fam**	**Pt**. **no**.	**Eth**	**Sex**	**Age**	**Mutation**	**Protein**	**Atypical hematological features**	**Bone marrow**	**Vascular features**	**Cardiac features**	**Renal and genital defects**	**Other features**	**Ref**
1	1	Pak	F	10	c.[130C > T]; [130C > T]	p.[P44S]; [P44S]	None	Normal cellularity and neutrophil maturation.	None	None	None	None	[13,21]
2	2	Pak	M	20	c.[130C > T]; [130C > T]	p.[P44S]; [P44S]	None	Normocellular with mild left-sided shift.	None	None	None	A single episode of myositis	[14,30]
3	3	Pak	M	10	c.[130C > T]; [130C > T]	p.[P44S]; [P44S]	None	Normocellular	None	None	None	None	[14]
4	4	Turk	M	18 m	c.[346A > G]; [346A > G]	p.[M116V]; [M116V]	T-cell lymphopenia, monocytosis and anemia.	Dysplastic	Primary PHT since infancy.	ASD	Bilateral inguinal herniae and undescended testes	Diagnosed with Dursun syndrome. Thymus hypoplasia, proximally placed thumbs, broad nasal bridge, pectus carinatum and high arched palate. Died at 18 m due to severe respiratory distress.	[12,18]
	5	Turk	F	18 m			Lymphopenia, monocytosis and anemia.	Dysplastic	Primary PHT since infancy.	ASD	None	Diagnosed with Dursun syndrome. Thymus hypoplasia, proximally placed thumbs, broad nasal bridge, pectus carinatum, single palmar creases and high arched palate. Died at 18 m due to severe respiratory distress.	[12,18]
5	6	NA	NA	NA	c.[347 T > A]; [347 T > A]	p.[M116K]; [M116K]	None	Not described	Prominent veins	ASD, mild mitral and tricuspid insufficiency	None	Learning difficulties and Hypogonadotrophic hypogonadism.	[26]
6	7	Pak	F	13	c.[347 T > C]; [347 T > C]	p.[M116T]; [M116T]	None	Normal cellularity and neutrophil maturation.	None	None	None	None	[13,21]
7	8	NA	F	NA	c.[353C > G]; [c.353C > G]*	p.[T118R]; [T118R]	None	Not described	Not described	ASD	Not described	Not described	[17]
8	9	Turk*	F	13	c.[461 T > C]; [461 T > C]*	p.[L154P]; [L154P]	None	Hypercellular marrow, myeloid hyperplasia, no maturation arrest.	Prominent veins on neck, chest, and abdomen.	Mild mitral regurgitation	None	Frontal bossing, depressed nasal bridge, upturned nose and retrognathia. Learning difficulties. Poorly developed secondary sexual characteristics. Elevated serum total cholesterol and LDL cholesterol.	[27]
9	10	Turk	F	12	c.[554 T > C]; [554 T > C]	p.[L185P]; [L185P]	None	Not described	Prominent veins	ASD and pulmonary valve stenosis.	None	None	[9]
10	11	Turk	F		c.[623 T > G]; [623 T > G]	p.[L208R]; [L208R]	None	Not described	None	PFO and tricuspid insufficiency.	None	None	[14]
11	12	Arab	F	12	c.[758G > A]; [758G > A]	p.[R253H]; [p.R253H]	None	Reduced mature neutrophils.	Prominent veins	ASD and small PDA.	Right grade III VUR. Discontinuous labia majora and minora.	Close set, down sloping eyes, low set ears	[11]
12	13	Arm, Turk	M	6	c.[758G > A]; [758G > A]	p.[R253H]; [p.R253H]	None	Reduced mature neutrophils.	Prominent veins	ASD	Bilateral cryptorchidism	None	[9]
	14	Arm, Turk	F	3			None	Reduced mature neutrophils.	Prominent veins and pulmonary venous anomaly.	Cor triatriatum	None	None	[9]
	15	Arm, Turk	F	11			None	Not described	Prominent veins	ASD and mitral insufficiency.	None	None	[9]
	16	Arm, Turk	M	6			None	Reduced mature neutrophils.	Prominent veins	ASD	Bilateral cryptorchidism	None	[9,30]
13	17	Arm, Turk	M	4	c.[758G > A]; [758G > A]	p.[R253H]; [p.R253H]	None	Reduced mature neutrophils.	Prominent veins	Not reported	Not reported	Poor growth	[9]
14	18	Arab, Israel	F	29	c.[758G > A]; [758G > A]	p.[R253H]; [p.R253H]	None	Hypercellular marrow with myeloid hyperplasia. Increased blast-like forms and megakaryocytes including atypical forms.	Prominent veins and varicose veins.	None	None	SGA, mild learning difficulties. Hypothyroidism. Dyslipidemia and elevated pancreatic amylase and uric acid.	[10]
	19	Arab, Israel	M	26			None	Mildly decreased myeloid cells. Increased megakaryocytes.	Prominent veins and varicosities.	None	None	SGA, FTT and mild learning difficulties. Hypothyroidism and secondary sexual characteristics.	[10]
	20	Arab, Israel	F	25			None	Hypercellular marrow. Mild dysmyelopoetic changes. Decreased erythropoiesis.	Prominent veins and varicosities.	ASD and PDA	None	Poor growth, mild learning difficulties and delayed menarche.	[10]
	21	Arab, Israel	M	2			Monocytosis, lymphopenia.	Normocellular	Prominent veins on face. PHT.	ASD, PDA and critical PS.	Bilateral crytorchidism	Liver calcifications. Mild learning difficulties. Pectus excavatum. Raised gamma glutamyltranspeptidases.	[10]
15	22	Cauc	M	9	c.[778G > C]; [778G > C]	p.[G260R]; [G260R]	None	Not described	Prominent veins	ASD	Micropenis	Mild developmental delay. Hypoplastic nipples, malar flattening, bilateral metatarsus adductus and thick erythematous skin on palms and soles.	[11]
16	23	Cauc	M	11	c.[778G > C]; [778G > C]	p.[G260R]; [G260R]	None	Reduced mature neutrophils.	Prominent veins	PDA, ASD, bicuspid aortic valve.	Cryptorchidism andmicropenis	Mild developmental delay. Clubbing. Palmar erythema, inverted nipples and malar flattening.	[11]
17	24	White Ger	F	7	c.[778G > C]; [778G > C]	p.[G260R]; [G260R]	None	Not described	Prominent veins	ASD	Urachal fistula	Microcephaly	[9]
18	25	White Ger	M	17	c.[778G > C]; [778G > C]	p.[G260R]; [G260R]	None	Not described	Prominent veins	ASD	Bilateral cryptorchidism and genital dysplasia	Growth retardation	[9]
19	26	NA	M	NA	c.[778G > C]; [778G > C]	p.[G260R]; [G260R]	None	Not described	Prominent veins	ASD	Bilateral cryptorchidism and genital dysplasia	Hypogammaglobunemia, microcephaly	[26]
20	27	Cauc	M	13	c.[778G > C]; [778G > C]	p.[G260R]; [G260R]	Iron deficiency anaemia	Pyknotic neutrophils, no maturation arrest. Myelokathexis. Emperipolesis of neutrophils in 12% of megakaryocytes. Atypical mononuclear megakaryocytes	Prominent veins. Mild PHT.	PFO, thickened mitral valve and dilated right ventricle.	Bilateral cryptorchidism, unilateral testicular agenesis, testicular microlithiasis and inguinal hernia.	Poor growth, microcephaly. Buffalo hump, ligamentous laxity, mild proximal muscle weakness and mild sensorineural hearing loss.	[20]
	28	Cauc	F	9			Iron deficiency anaemia	Pyknotic Neutrophils, no maturation arrest. Myelokathexis. Emperipolesis of neutrophils in 12% of megakaryocytes. Atypical mononuclear megakaryocytes	Prominent veins	Large ASD	None	Poor growth, microcephaly, ligamentous laxity, mild proximal muscle weakness and mild sensorineural hearing loss.	[20]
21	29	Turk	M	?	c.[779G > A]; [779G > A]	p.[G260D]; [G260D]	None	Maturation arrest at myelocyte/promyelocyte stage	Prominent veins	ASD	Left grade II hydronephrosis	Triangular face, frontal bossing, micrognathia and depressed nasal bridge. Cutis laxa. Bilateral hearing loss. Growth retardation.	[11]

**Table 2:** **Patients with homozygous G6PC3 truncating**, **frameshift and splice**-**site mutations**

**Fam**	**Pt. no.**	**Eth**	**Sex**	**Age**	**Mutation**	**Protein**	**Atypical hemat features**	**Bone marrow**	**Vascular features**	**Cardiac features**	**Renal and genital defects**	**Other features**	**Ref**
22	30	White Greek	F	13	c.[141C > G]; [141C > G]	p.[Y47X]; [Y47X]	None	Not described	Prominent veins	None	None	None	[9]
23	31	Arab, Leb	M	5	c.[144C > A]; [144C > A]	p.[Y48X]; [Y48X]	None	Not described	No	None	Cryptorchidism and bilateral inguinal hernia.	Cleft palate	[9]
24	32	Pak	M	28	c.[190_210del]; [190_210del]	p.[T64_I70del]; [T64_I70del]	None	Not described	None	ASD	None	Granulomatous inflammatory bowel disease, splenomegaly, digital clubbing and short stature.	[14,30]
24	33	Pak	F	16			None	Not described	None	None	None	Granulomatous inflammatory bowel disease, splenomegaly, digital clubbing and short stature.	[14,30]
25	34	Hisp	F	12	c.[210delC]; [210delC]	p.[I70fsX46]; [I70fsX46]	None	Maturation arrest at myelocyte/promyelocyte stage	Prominent veins	Small ASD	None	Triangular face, depressed nasal bridge, growth retardation, growth hormone deficiency, enlarged anterior pituitary lobe.	[11]
26	35	Hisp	M	14	c.[210delC]; [210delC]	p.[I70fsX46]; [I70fsX46]	None	Maturation arrest at myelocyte/promyelocyte stage	Prominent veins	ASD	None	Triangular face, depressed nasal bridge, osteoporosis, Kawasaki’s disease, growth retardation and delayed puberty.	[11]
27	36	NA	M	NA	c.[210delC]; [210delC]	p.[I70fsX46]; [I70fsX46]*†	None	Not described	Not described	ASD and coronary aneurysm	Not described	Not described	[17]
28	37	Hisp	M	9	c.[218 + 1G > A]; [218 + 1G > A]	p.[?];[?]	None	Reduced mature neutrophils and increased reticular staining.	Prominent veins	ASD	Right inguinal hernia. Bilateral cryptorchidism.	Frontal bossing upturned nose, recessed chin and triangular face.	[11]
29	38	Mor	M	22	c.[257delA]; [257delA]*	p.[E86fs]; [E86fs]	Iron deficiency anaemia	Maturation arrest at promyelocyte stage	Prominent veins	ASD	Bilateral cryptorchidism	Poor growth	[38]
30	39	Persian	M	1	c.[416G > T]; [416G > T]	p.[?];[?]* ‡	None	Maturation arrest at myelocyte/promyelocyte stage	Prominent veins	ASD	None	None	[11]
31	40	Persian	M	9 m	c.[416G > T]; [416G > T]*	p.[?];[?]* ‡	None	Maturation arrest inmyelocyte stage	Not reported	ASD	None	Failure to thrive. Died at 9 m due to severe lung infection.	[37]
32	41	Hisp	M	11	c.[766_777del]; [766_777del]	p.[S255fs]; [S255fs]	Mild normocytic anaemia	Maturation arrest at myelocyte/promyelocyte stage	Prominent veins	ASD, MR and TR.	Left inguinal hernia. Right cryptorchidism.	Broad face, prominent ears, small nose, big mouth, narrow forehead and short philtrum. Mild hepatomegaly. Bilateral inner ear hearing loss.	[11]
33	42	Pak	F	1	c.[766_777del]; [766_777del]	p.[S255fs]; [S255fs]	None	Reduced mature neutrophils	None	Hypoplastic left ventricle (mild)	None	Congenital ptosis and growth retardation.	[11]
34	43	Hisp	M	19	c.[766_777del]; [766_777del]	p.[S255fs]; [S255fs]	Anaemia	Reduced mature neutrophils and magakaryocyte hyperplasia.	Prominent veins	ASD, MR and TR.	Right cryptorchidism.	Sensorineural hearing loss and prominent ears.	[28]
35	44	Irish	F	38	c.[829C > T]; [829C > T]	p.[Q277X]; [Q277X]	None	Hyperplasia of granulocyte precursors with maturation arrest	Varicose veins and PHT at 35y	ASD	Ureteric re-implantation at 12 months	Crohn's disease diagnosed at 7y, midface hypoplasia, full lips and prognathism.	[22,23]
	45	Irish	M	37			None	Not described	None	Mitral valve prolapse	Ureteric re-implantation at 2y	Crohn's disease diagnosed at 15y, mid-face hypoplasia, full lips and prognathism. Died at 37y due to multi-organ failure following infective endocarditis.	[22]
36	46	Persian	F	11	c.[935dupT]; [935dupT]	p.[N313fs]; [N313fs]	None	Maturation arrest atmyelocyte/promyelocyte stage	None	ASD	Mild VUR at 7 m (resolved at 2y).	Triangular face, depressed nasal bridge, growth retardation and cutis laxa.	[11]
37	47	Persian	M	2y	c.[935dupT]; [935dupT]	p.[N313fs]; [N313fs]	None	Maturation arrest inmyelocyte stage	Prominent veins	ASD	Unilateralhydronephrosis	None	[37]
38	48	Persian	M	10	c.[935dupT]; [935dupT]	p.[N313fs]; [N313fs]	None	Not described	No	ASD and PDA	None	None	[9]

**Table 3:** Patients with compound heterozygous G6PC3 deficiency

**Fam**	**Pt. no.**	**Eth**	**Sex**	**Age**	**Mutation**	**Protein**	**Atypical hemat features**	**Bone marrow**	**Vascular features**	**Cardiac features**	**Renal and genital defects**	**Other features**	**Ref**
39	49	Cauc	M	7	c.[131C > T]; [758 G > A]	p.[P44L]; [R253H]	None	Reduced numbers of mature neutrophils	Prominent veins	ASD	Bilateral undescended testis, poor renal cortico-medullary differentiation	Flat malar region, short philtrum, splenomegaly, right ptosis	[11]
40	50	Cauc	M	7	c.[208insC]; [778G > C]	p.[I70fsX16]; [G260R]	None	Hypocellular bone marrow with mature arrest.	Prominent veins	ASD	Left inguinal hernia	Triangular face, height and weight below 3rd centile and growth hormone deficiency.	[11]
41	51	Hisp	M	1	c.[210delC]; [348G > A]	p.[I70fsX46]; [M116I]	None	Maturation arrest at promyelocyte/myelocyte stage	Prominent veins	ASD	Ambiguous genitalia. Enlarged prostatic utricle and congenital hydronephrosis.	Triangular face, prominent upper lip, depressed tip of nose, narrow thorax, inverted nipples and flattening of acetabulum with the sacroiliac notch.	[11]
42	52	NA	F	18	c.[326–1 G > A]; [778G > C]	p.[?]; [Gly260R]	T-cell lymphopenia	Normal haematopoiesis	Prominent veins	Mitral valve insufficiency	None	Inflammatory bowel disease diagnosed at 8y, hypergammaglobulinemia and growth delay.	[19]
43	53	Cauc	M	18	c.[482G > A]; [565C > T]	p.[R161Q]; [R189X]	None	Not described	Prominent veins	ASD and bicuspid aortic valve	Small kidneys, bilateral undescended testes	Delayed puberty, Pyloric stenosis neonatally, massive splenomegaly age 14 years requiring removal, growth retardation.	[11]
44	54	Cauc	F	16	c.[677 + 1G > A]; [829C > T]	p.[?]; [Gln277X]	None	Not described	Prominent veins	ASD	None	None	[11]
45	55	White French	F	13	c.[677 + 1G > A]; [829C > T]	p.[?]; [Gln277X]	None	Not described	Prominent veins	None	None	Myopathy	[9]
46	56	White British	F	8	c.[757C > T]; [1000_1001del]	p.[R253C]; [M334fs]	None	Normal cellularity and maturation.	None	None	None	None	[13]
	57	White British	F	18			None	Not described	None	None	None	None	[13]

Key for Tables 1, 2 and 3: *ASD* atrial septal defect, *Arm* Armenian, *Cauc* Caucasian, *Eth* ethnicity, *Fam* family, *Ger* German, *hemat* haematological, *Hisp* Hispanic, *Leb* Lebanese, *Mor* Moroccan, *MR* mitral regurgitation, *NA* not available, *Pak* Pakistani, *PFO* patent foramen ovale, *PHT* pulmonary hypertension, *PS* pulmonary stenosis, Pt. no. – patient number, *Ref* references, *TR* tricuspid regurgitation, *Turk* Turkish, *VUR* vesico uretric reflux; and * denotes entries where the information is derived or corrected from the original publication. †The c.210delC mutation was described to result in p.I70fsX115 by Xia et al. However, the correct predicted protein with this mutation should be p.I70fsX46. ‡ Please see main text for explanation for how this mutation affects the splice site.

Patient numbers are continuous from Tables 1, 2 and 3.

### Clinical description

#### Typical haematological features

The haematological phenotype of G6PC3 deficiency is variable but severe neutropenia in peripheral blood (neutrophils count below 0.5×10^9^/L) is present in all reported patients. Patients with G6PC3 deficiency usually present in the first few months of life with recurrent bacterial infections although a patient presenting with symptoms only in late teenage years has been described [13]. Sino-pulmonary infections, otitis media, urinary tract infections, skin abscesses and sepsis are common. Some patients may also suffer from oral ulcers, periodontitis, stomatitis, gingivitis and fungal infections. Untreated patients remain susceptible to bacterial infections. Nearly two-thirds of patients with G6PC3 deficiency demonstrate intermittent thrombocytopenia [11], but this has not been reported to cause clinical consequences. Importantly, unlike some other constitutional neutropenia syndromes, no cases with myelodysplastic syndrome have been yet reported in untreated or G-CSF treated patients with G6PC3 deficiency. A systematic analysis has shown this to be statistically significant [11].

#### Atypical haematological features

In addition to the key haematological features described above, some patients with G6PC3 deficiency can present with additional haematological abnormalities. For example, a sibling pair who were originally described as the first cases of Dursun syndrome and later shown to have G6PC3 deficiency, had severe lymphopenia and thymic hypoplasia [12,18]. Another patient recently described by Bégin et al. had persistent lymphopaenia [19]. Interestingly, this patient had low naïve CD4^+^ counts suggesting a defect in thymic structure or function. G6PC3 deficiency, therefore, is in the differential diagnosis for Di-George (velo-cardio-facial) syndrome.

Bone marrow examination may show maturation arrest in the myeloid lineage but some patients with G6PC3 deficiency may have a hyper- or normo-cellular bone marrow [20,21]. The reason for this variability in the phenotype of G6PC3 deficiency is not clear [21] but it emphasises the importance of considering G6PC3 deficiency as a possible cause of neutropenia even if the bone marrow does not show the maturation arrest of the myeloid cell line.

#### Frequent non-haematological features

The majority of patients described with G6PC3 deficiency have additional non-haematological features that, in a clinical setting, help to distinguish G6PC3 deficiency from other causes of neutropenia. Tables 1, 2 and 3 summarize important clinical features of all 57 patients with G6PC3 deficiency described in the medical literature.

A prominent superficial venous pattern is the most easily clinically recognizable non-haematological feature of G6PC3 deficiency. At least 38/57 (66.6%) patients in the medical literature have been described to demonstrate this. We have shown that the superficial veins may be less evident in infancy in some patients and tend to gradually become more prominent with age [10]. In adults these vascular changes can develop into varicose veins and result in venous ulcers.

Forty four (77.1%) patients with G6PC3 deficiency have been reported to have a congenital cardiac anomaly, making it the most common non-haematological feature of the condition. Of these 44 patients, 37 had an atrial septal defect. Other rare heart anomalies include patent foramen ovale, cor triatriatum, patent ductus arteriosus, critical pulmonary stenosis and hypoplastic left ventricle. A range of valvular anomalies, including mitral valve prolapse, insufficiency or regurgitation, tricuspid insufficiency, bicuspid aortic valves and pulmonary valve stenosis has been described.

Twenty five (43.8%) patients with G6PC3 deficiency have been described to have renal system malformation or genital anomalies. These are significantly more common in male patients (16/31) than in females (4/25). The commonest problem in males is cryptorchidism (15/25). Other features in males include inguinal hernia, ambiguous genitalia, genital dysplasia, micropenis, poor renal cortico-medullary differentiation, hydronephrosis, small kidneys and vesico-uretric reflux. In females, vesico-uretric reflux, urachal fistula and genital dysplasia have been described.

#### Rare non-haematological features

At least five patients, including two sibling pairs, with G6PC3 deficiency have been described with inflammatory bowel disease (IBD) [14,19,22,23]. This is in the context of a background prevalence of IBD of one in 250 in the UK [24]. IBD in G6PC3 deficiency most closely resembles Crohn’s disease and the intestinal pathology of GSD1b [19]. IBD is more commonly present in individuals with other causes of neutropenia and Fernandez et al. therefore suggested that this feature may be secondary to the neutropenia [22].

Pulmonary hypertension (PHT) has been described in 5/57 patients from three families. The diagnosis of increased pulmonary arterial pressures, unrelated to congenital heart defect, was made in infancy in one patients with G6PC3 deficiency [10] and in a sib-pair who were initially described as Dursun syndrome [12,18]. In two other patients, PHT was noted only in later life [20,22]. Interestingly, PHT is also a well recognized feature of GSD1 [25].

Endocrine abnormalities have been described in 8/57 patients with G6PC3 deficiency. These include growth hormone deficiency in two patients, including one with an enlarged anterior pituitary lobe [11]. Delayed puberty, hypogonadotropic hypogonadism or poorly developed secondary sexual characteristics have been noted in six patients [10,11,26,27]. Additionally, hypothyroidism was noted in an affected brother and sister [10].

Intrauterine growth retardation, failure to thrive and poor postnatal growth have been described in a number of children. It is not clear if the postnatal growth retardation is part of the primary phenotype or secondary to repeated infections. Notably, female g6pc3^−/−^ mice exhibit growth retardation [8].

Some children have also been described with minor facial dysmorphism, mainly with a triangular face and a depressed nasal bridge. Experience with adult phenotype is limited but fullness of face may be an additional feature [10,23]. Minor skeletal and integument anomalies have been described in a number of patients [10]– [12]. These include scoliosis, pectus carinatum, ligamentous laxity, loose skin or cutis laxa, palmar erythema, small and/or inverted nipples. Neurological and muscular features such as microcephaly [9,20,26], sensori-neural hearing loss [11,20,28], myopathy [9], myositis [14], muscle weakness [20], and congenital ptosis [11] have been described in some patients. Cleft palate or high palate has also been described rarely [9].

One family included four patients with G6PC3 deficiency with mild to moderate developmental delay and learning difficulties [10]. Intellectual deficits have not been observed in any other patients with G6PC3 deficiency and could have been coincidental to the G6PC3 deficiency in this family. In the same family one child had raised gamma-glutamyl transferase, liver calcifications and choledocholithiasis and one adult patient had a low HDL levels and increased amylase activity. Raised serum cholesterol and LDL levels were recently noted in one patient [27].

#### Non-syndromic congenital neutropenia

At least six individuals with G6CP3 deficiency have been described with isolated neutropenia without any other manifestations of the disease. This suggests that more than 10% cases of G6CP3 deficiency could be apparently non-syndromic, although even this could be an underestimate due to ascertainment bias. This observation demonstrated that G6PC3 deficiency should be considered as part of the differential diagnoses in any patient with unexplained congenital neutropenia.

### Aetiology and disease mechanism

**Figure 2:**
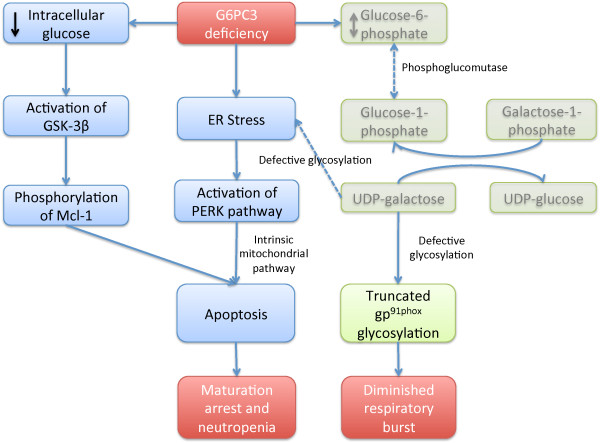
**A flow diagram summarising proposed mechanisms of haematological features of G6PC3 deficiency.** G6PC3 deficiency leads to decreased cytoplasmic glucose and glucose-6-phosphate levels [31] and ER stress and activation of protein like ER-kinase (PERK) [9,29]. The lower levels of glucose possibly lead to activation of GSK-3β and phosphorylation of Mcl-1. Activation of these pathways contributes to apoptosis of the cells (this part of the pathway is shown in blue boxes). G6PC3 deficiency also results in aberrant glycosylation of a NADPH oxidase subunit, gp91phox (shown in green box). The precise mechanism of aberrant glycosylation is not clear but may be mediated by perturbation of the Leloir pathway of galactose metabolism (shown in faded green). The final effect of these dysfunctions is maturation arrest of neutrophils, neutropenia and diminished respiratory burst (shown in red boxes).

Mutations in *G6PC3* result in significantly reduced enzyme activity [9]. Bone marrow promyelocytes of patients with G6PC3 deficiency show increased expression of BiP, suggesting ER stress (Figure 2) [9]. Consistent with this, activation of the protein kinase-like ER kinase (PERK) pathway has been demonstrated in neutrophils of g6pc3^−/−^ mice [29]. Neutrophils and skin fibroblasts of patients with G6PC3 deficiency have increased rates of apoptosis after induction with TNF-α or agents that induce ER stress [9]. The apoptosis is mediated, in part, by the intrinsic mitochondrial pathway [29]. G-CSF modulates these apoptotic mediators and leads to increased glucose uptake, elevated intracellular levels of glucose-6-phosphate, lactate, and adenosine-5’-triphosphate in neutrophils. Additionally, decreased intracellular glucose concentration could activate GSK-3β, leading to phosphorylation of Mcl-1 and induction of apoptosis (Figure 2).

In addition to the decreased neutrophil numbers, dysfunction of neutrophil activity has also been demonstrated in some patients. Hayee et al. showed severe defects in the synthesis of N- and O- glycans in patients with G6PC3 deficiency (Figure 2) [30]. Notably, they showed truncated N-glycosylation of gp91phox, a component of nicotinamide adenine dinucleotide phosphate (NADPH) oxidase found in secondary granules of neutrophils. They proposed that aberrant glycosylation might underlie the diminished respiratory burst seen in G6PC3 deficient granulocytes and hypothesized that the glycosylation defects may also contribute towards ER stress and apoptosis. The mechanism of aberrant glycosylation is unclear. Hayee et al. postulated that accumulation of glucose-6-phosphate in G6PC3 deficiency might inhibit formation of UDP-galactose through the Leloir pathway of galactose metabolism. However, Jun et al. have shown lower levels of glucose-6-phosphate and lower glucose uptake in cells of *g6pc3*^−/−^ mice [31].

Some patients with G6PC3 deficiency paradoxically demonstrate normal or hypercellularity of the bone marrow. McDermott et al. studied such a sibling pair and showed increased expression of CXCR4 in neutrophils [20]. They proposed that in such patients, stress induces overexpression of CXCR4 in patients leading to reduced egression or premature return of granulocytes to the bone marrow for destruction. They also showed in *g6pc3*^−/−^ mice that a specific CXCR4 antagonist, AMD3100, rapidly reversed neutropenia.

The immune problems of G6PC3 deficiency extend beyond neutrophil dysfunction. Jun et al. demonstrated impaired macrophage respiratory burst, chemotaxis, calcium flux and phagocytic activities with down-regulation of reduced NADPH oxidase subunits and membrane translocation of p47phox, [31].

### Molecular diagnosis and the mutational spectrum of G6PC3

### Summary of mutations

*G6PC3* maps to 17q21.31, consists of six exons and encodes the G6PC3 protein that is comprised of 346 residues and is anchored in the ER by nine transmembrane helices in a way that keeps the active site inside the ER lumen [5,6]. Histidine at position 167 is the phosphate acceptor site and arginine at 79 and histidine at 114 are proton donors [32]. The signature phosphatase motif, K-X_6_-RP-(X_12-54_)-PSGH-(X_31-54_)-SR-X_5_-H-X_3_-D, spans between residues 66 and 171 of G6PC3.

**Figure 3:**
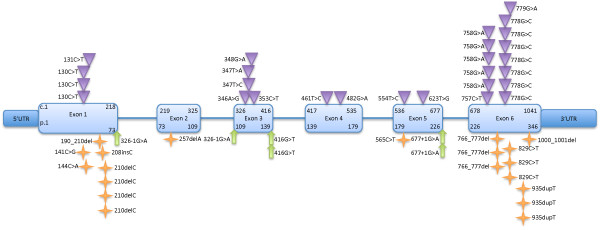
**A summary of *G6PC3 *mutations.** A schematic representation of the G6PC3 gene. The starting and ending nucleotide numbers of the cDNA and their corresponding amino acid residue numbers are provided within each exon. Each mutation is represented once for every family in which it was detected. The inverted triangles represent missense mutations, the block-arrows represent splice-site mutations and stars represent truncating or frame-shift mutations. UTR is untranslated region.

G6PC3 deficiency can be diagnosed by sequencing all the six exons of the gene (NM_138387.3). There are now at least 57 patients with genetically proven G6PC3 deficiency described in the literature (Tables 1, 2 and 3). Of the 114 mutated alleles, 66 are missense (Figure 3). Fourteen *G6PC3* missense mutations resulting in amino acid substitutions in nine different codons have been identified. These missense mutations are spread all across the gene but no missense mutation has been described in exon 2. The largest number of mutated missense alleles have been described in exon 6. The other 48/114 mutated *G6PC3* alleles result from four nonsense mutations, seven frameshift deletions or insertions and four splice-site mutations.

Of note, the c.416G > T mutation has been identified in two patients and predicted to be a missense change (p.S139I) [11,27]. However, the codon for residue S139 is determined by the last two nucleotides of exon 3 and the first nucleotide of exon 4. The nucleotide change responsible for p.S139I is c.416G > T and is the last nucleotide of exon 3. Hence, it is possible that this change, in fact, affects splicing. The resultant protein has not been characterized and NNsplice software predicts the spice donor site in the wild-type sequence is lost with c.416G > T [33]. We have, therefore, considered c.416G > T as a splice-site mutation in our analysis.

### Founder mutations

To date, a number of *G6PC3* mutations have been identified more frequently in particular ethnic groups. The homozygous p.P44S mutation has been described in three unrelated Pakistani patients and in two of these patients it was confirmed to be present on a common haplotype [14]. Ten patients, from four unrelated families, with the homozygous p.R253H mutation have been ascertained from different countries in the Middle East, suggesting that p.R253H may be a founder mutation in this population. Seven patients from six families with the homozygous p.G260R mutation have a White European ancestry. Two patients with compound heterozygous mutations also have p.G260R as one of the changes suggesting that it is an ancient European founder mutation in Caucasians. Other mutations described in multiple unrelated patients from particular ancestry include p.G277X in Europeans, p.N313fs in Persians and p.I70fsX46 in Hispanic patients.

### Functional prediction of *G6PC3* mutations

The mechanism by which *G6PC3* missense mutations lead to loss of enzyme activity remains unclear. Notably, the protein structure of G6PC3 has not been defined. *G6PC3* is a paralog of *G6PC* and the two genes, along with *G6PC2*, are thought to have arisen from ancient duplication events [34]. The structure of liver glucose-6-phosphatase (coded by *G6PC*) is better characterized [35]. Due to their similarities, G6PC may serve as a useful model for studying G6PC3 [5,6]. The G6PC molecule is constituted from 357 amino acids (in contrast with 346 for G6PC3) and 120 of 346 (~35%) amino acids are identical between the two molecules.

**Figure 4:**
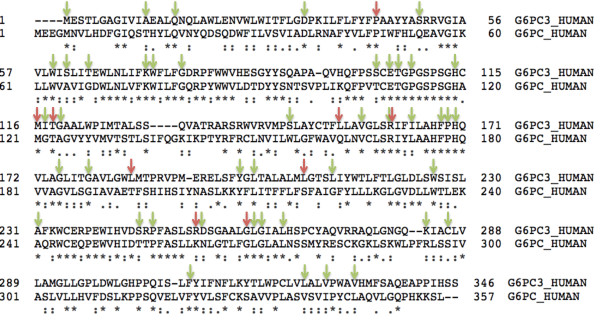
**G6PC3 and G6PC alignment and known missense mutations.** A ClustalW alignment of G6PC3 and G6PC amino acid sequences. Identical residues are marked by *. Sites where missense mutation has been identified in G6PC3 are highlighted by red arrows. Green arrows highlight missense mutations in GSD1a. The conserved residues are enriched in missense mutations described in two diseases.

**Figure 5:**
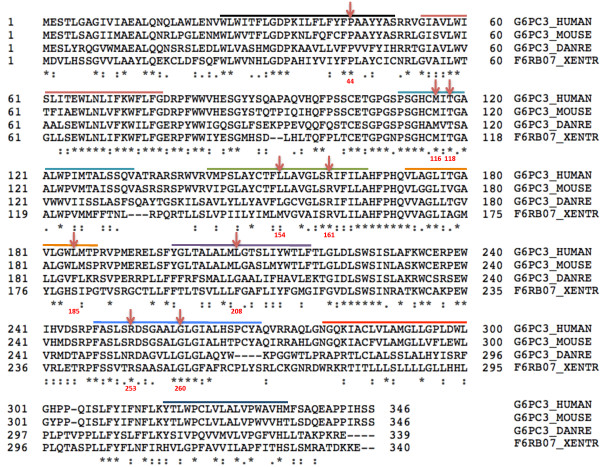
**G6PC3 multi**-**species alignment.** Multi-species ClustalW alignment of G6PC3. Identical residues are marked by *. Sites where missense mutation has been identified in G6PC3 are highlighted by red arrows. Amino acids in trans-membrane regions are shown under lines of different colours. Note that all the missense mutations affect trans-membrane residues.

Out of the nine sites substituted in patients with G6PC3 deficiency, five amino acids (P44, M116, T118, R161 and G260) demonstrate complete conservation between G6PC3 and G6PC amino acid sequences (Figure 4). One residue, R253, is semi-conserved and three are not conserved (L154, L185 and L208). Furthermore, three *G6PC3* mutants with reduced enzyme activity that have not been demonstrated in patients, but were created in the laboratory (R79A, H114A and H167A), also affect fully conserved sites [32]. Six out the nine residues affected by missense *G6PC3* mutations are fully conserved in orthologs till the level of *Xenopus* (P44, M116, T118, R161, R253 and G260) (Figure 5). L154 is conserved till *Drosophila* and L185 and L208 are identical in human and mouse. All the active site residues are fully conserved.

In GSD1a, 54 *G6PC* missense mutations resulting in substitution of 48 residues have been reported [36]. Out of these 48 residues, 29 are completely conserved between G6PC and G6PC3 (Figure 4). Combining the *G6PC* and *G6PC3* missense mutations data, 33 of 120 highly conserved and only 20 of 226 semi-conserved or non-conserved residues are substituted due to a missense mutation in either GSD1a or G6PC3 deficiency. This difference is statistically significant (p < 0.0001, calculated by Fisher’s exact test). Hence, it is likely that the protein structure, the mutation spectrum and the molecular mechanisms underlying missense mutations in the two diseases are comparable.

*G6PC* missense mutations have been classified into those affecting active site, helical and non-helical depending on their predicted topological position [36]. Studies have demonstrated that active site mutations abolish the G6PC enzyme activity without affecting its stability. The majority of the helical substitutions destabilize the mutant protein because the integrity of trans-membrane helices is critical for correct folding and stability. Moreover, the non-helical regions of G6PC have been shown to be less important for enzyme stability and activity.

**Figure 6:**
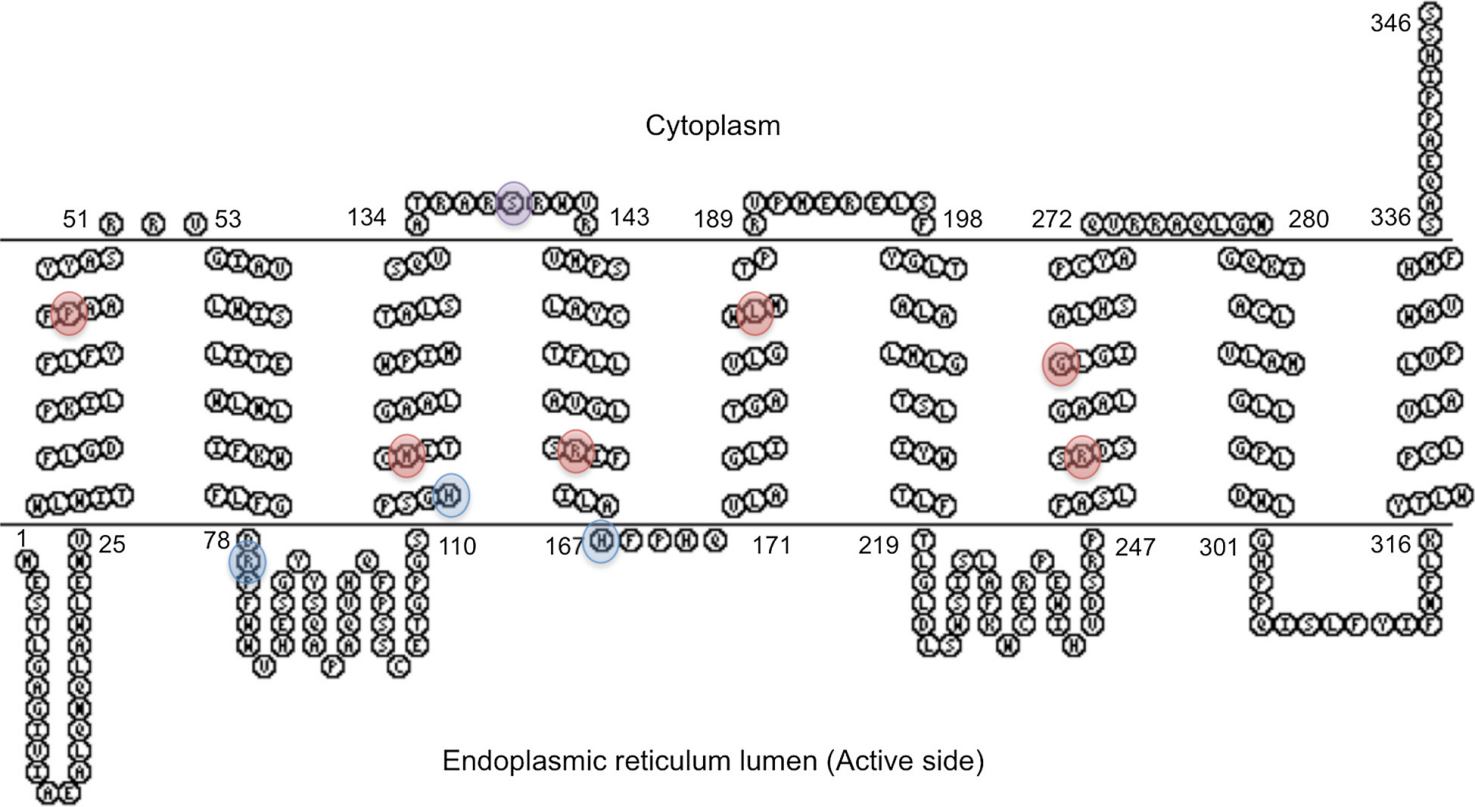
**Predicted topology of G6PC3 with missense mutations.** Residues substituted by missense mutation are highlighted by red circles and the laboratory generated substitutions by blue circles. The S139 residue, predicted in previous publications to be substituted by c.416G > T mutation is highlighted by purple circle. Our analysis suggests that the c.416G > T might be a splice-site mutation (see main text for discussion).

Mapping known missense *G6PC3* mutations on the predicted protein structure shows that all nine missense mutations are predicted to be helical (Figure 6). Hence analogous to findings in G6PC, we predict the nine known *G6PC3* mutations result in decreased enzyme stability and loss of function.

The mutation data additionally highlights the importance of certain critical G6PC3 residues. For example, four different substitutions affecting residue number 116 (p.M116V, p.M116K, p.M116T and p.M116I) have been reported in five patients with G6PC3 deficiency (Figure 3). Similarly, two different substitutions have been detected affecting residue 44 in three patients (p.P44S and p.P44L), with p.P44S detected in two unrelated Pakistani patients.

Truncating mutations can result in nonsense-mediated decay of the transcript, or in formation of a non-functional aberrant protein. In autosomal recessive disorders, therefore, patients homozygous for truncating mutations are predicted to have a complete absence of the protein encoded by the mutated gene. At least 19 patients with bi-allelic truncating *G6PC3* mutations (excluding splice-site mutations) have been described to date (Table 2). If mutations in these patients result in complete absence of G6PC3 enzyme activity, then clearly it is compatible with life in humans. However, studies of enzyme activity have not yet been reported in any patients with truncating G6PC3 mutations. Moreover, current evidence does not indicate that patients with bi-allelic truncating *G6PC3* mutations have a more severe phenotype than most patients with missense *G6PC3* mutations. Another possibility is that an alternative in-frame translation start site could be used in patients with truncating mutations. In this context, it is interesting to note that apart from p.E86fs, all truncating *G6PC3* mutations cluster in in exons 1, 5 or 6, which flank the exons coding for the phosphatase activity. This is in contrast with *G6PC3* missense mutations that are spread across all exons. Without evidence from functional work it is impossible to conclude if this observation has a functional basis or if it is an incidental finding.

The truncating mutation closest to the carboxy-terminal of the G6PC3 protein is p.M334fs. This mutation has not been characterised functionally but it is predicted to result in read through of additional amino acids and not cause premature termination of the protein. Exploration of the mechanism in this mutation could provide insight about the role of terminal residues of the protein.

### Genotype-phenotype correlation in G6PC3 deficiency

We have shown absence of a genotype–phenotype relationship for the bone marrow phenotype in patients with G6PC3 deficiency [21]. However, our recent work has suggested that the extended phenotypic spectrum may depend on the genotype of the patients [13]. Specifically, certain mutations e.g. p.P44S seem to be more often associated with non-syndromic neutropenia with absence of other systemic features. This hypothesis is based on a small number of patients and additional patients need to be identified to test it.

### Differential diagnosis

Congenital neutropenia is a genetically and phenotypically heterogeneous group of conditions. Broadly, these conditions can be divided into those where neutropenia is the predominating feature, such as in *ELANE*, *GFI1*, *HAX1*, *CSF3R* and *WAS* mutations and those where neutropenia is part of a multi-system disorder. The later group includes glycogen storage disorder type 1b, Schwachman-Diamond syndrome, WHIM syndrome, Barth’s disease, Chediak-Higashi syndrome, Hermansky-Pudlak syndrome type 2, Griscelli syndrome type 2, P14 deficiency, Clericuzio poikiloderma with neutropenia, Pearson syndrome, Cohen syndrome, cartilage-hair hypoplasia and Charcot-Marie-Tooth disease type 2 M [16].

### Management

Neutropenia of a number of patients with G6PC3 deficiency has been treated with G-CSF. It leads to improvement in neutrophil numbers, prevents infections and improves quality of life [9,11,20]. However, in some patients G-CSF may fail to control infections even in large doses [14]. On the other hand, mildly affected patients may not necessarily require G-CSF treatment and can be managed with prophylactic antibiotics. T-cell subsets may be examined if accompanying cellular immunodeficiency is suspected.

Echocardiogram, renal and pelvic ultrasound scans should be performed in all cases of suspected or confirmed G6PC3 deficiency. Expert advice in management of congenital heart defects and genito-urinary problems must be sought. Patients, especially adults should be monitored for development of varicose veins and venous ulcers. Growth parameters should be monitored in all children with G6PC3 deficiency. If indicated, evaluation of growth hormone levels in children and pubertal development in adolescents should be considered. Biochemical profile monitoring could be part of routine part of the follow-up. Of note, raised total serum cholesterol and LDL levels were reported to normalize with statins in one patient [27]. Rare manifestations such as PHT and IBD should be managed according to standard guidelines. One patient with G6PC3 deficiency and IBD required a right hemi-colectomy and showed good response to anti-TNF treatment, infliximab [19]. The authors suggested considering bone marrow transplantation for patients with G6PC3-related IBD refractory to anti-TNFα therapy.

### Prognosis

Prognosis of most patients with G6PC3 deficiency is generally good on G-CSF treatment or prophylactic antibiotics in mildly affected individuals. However, untreated G6PC3 deficiency can be fatal. Both patients with Dursun syndrome died at 18 months due to severe respiratory distress [12,18]. The oldest patient ever described with G6PC3 deficiency died at 37 years of age due to infective endocarditis [22]. Of note this patient was non-compliant with his treatment. The youngest reported death in a patient with G6PC3 deficiency was at nine months due to severe lung infection [37].

### Unresolved questions

A number of questions remain to be answered in G6PC3 deficiency. Identification of more patients is required to clarify the extended phenotypic spectrum of the disease and the possible genotype-phenotype relationship. Although no cases of myelodysplastic syndrome have yet been noted in G6PC3 deficiency, long-term follow-up will be required to confirm this observation. At present, the reason for variability of the bone marrow phenotype in patients with mutations in G6PC3 is uncertain and further characterisation is warranted. Understanding the molecular basis of mutations leading to loss of enzyme activity may help to explore the therapeutic potential of small molecule chaperones in this disease, especially because missense mutations affect the majority of patients with G6PC3 deficiency. Furthermore, a similar approach may also benefit patients with GSD1a. Much work has been undertaken to understand the effect of G6PC3 deficiency on neutrophils, but mechanisms underpinning the developmental defects of other organs and other haematological lineages in the condition remain to be studied.

## Conclusions

In this review we have compiled the clinical features of all the 57 patients with G6PC3 deficiency described in the literature demonstrating the variability of the condition. This will help in management of patients and in genetic counselling of families. G6PC3 deficiency should be part of the diagnostic screen of any patient with severe congenital neutropenia. Analysis of mutational spectrum provides insights into the disease mechanism and reveals a possible genotype-phenotype relationship.

### Endnote

^a^Note – G6PC3 gene is incorrectly mapped to ORPHA486 (autosomal dominant severe congenital neutropenia) on http://www.orpha.net.

## Abbreviations

ER: Endoplasmic reticulum; IBD: Inflammatory bowel disease; G6PC3: Ubiquitous glucose-6-phosphatase; G-CSF: Granulocyte-colony stimulating factor; NADPH: Nicotinamide adenosine dinucleotide phosphate; PHT: Pulmonary hypertension.

## Competing interests

The authors have no competing interests to declare.

## Authors’ contributions

Both authors planned the study. SB compiled and reviewed the data and wrote the manuscript. WGN reviewed the manuscript. Both authors read and approved the final manuscript.

## Disease name/synonyms

G6PC3 deficiency^a^

Ubiquitous glucose-6-phosphatase deficiency

Severe congenital neutropenia type 4

Dursun syndrome (ORPHA178503)

## References

[B1] Hutton JC O’Brien RM Glucose-6-phosphatase catalytic subunit gene family J Biol Chem 2009 284 29241 29245 10.1074/jbc.R109.025544 19700406 PMC2785553

[B2] Lei KJ Shelly LL Pan CJ Sidbury JB Chou JY Mutations in the glucose-6-phosphatase gene that cause glycogen storage disease type 1a Science 1993 262 580 583 10.1126/science.8211187 8211187

[B3] Janecke AR Mayatepek E Utermann G Molecular genetics of type 1 glycogen storage disease Mol Genet Metab 2001 73 117 125 10.1006/mgme.2001.3179 11386847

[B4] Gerin I Veiga-da-Cunha M Achouri Y Collet J-F Van Schaftingen E Sequence of a putative glucose 6-phosphate translocase, mutated in glycogen storage disease type lb FEBS Letters 1997 419 235 238 10.1016/S0014-5793(97)01463-4 9428641

[B5] Guionie O Clottes E Stafford K Burchell A Identification and characterisation of a new human glucose-6-phosphatase isoform FEBS Letters 2003 551 159 164 10.1016/S0014-5793(03)00903-7 12965222

[B6] Shieh J-J Pan C-J Mansfield BC Chou JY A glucose-6-phosphate hydrolase, widely expressed outside the liver, can explain age-dependent resolution of hypoglycemia in glycogen storage disease type Ia Journal of Biological Chemistry 2003 278 47098 47103 10.1074/jbc.M309472200 13129915

[B7] Cheung YY Kim SY Yiu WH Pan CJ Jun HS Ruef RA Lee EJ Westphal H Mansfield BC Chou JY Impaired neutrophil activity and increased susceptibility to bacterial infection in mice lacking glucose-6-phosphatase-beta J Clin Invest 2007 117 784 793 10.1172/JCI30443 17318259 PMC1797608

[B8] Wang Y Oeser JK Yang C Sarkar S Hackl SI Hasty AH McGuinness OP Paradee W Hutton JC Powell DR O’Brien RM Deletion of the gene encoding the ubiquitously expressed glucose-6-phosphatase catalytic subunit-related protein (UGRP)/glucose-6-phosphatase catalytic subunit-β results in lowered plasma cholesterol and elevated glucagon J Biol Chem 2006 281 39982 39989 10.1074/jbc.M605858200 17023421

[B9] Boztug K Appaswamy G Ashikov A Schäffer AA Salzer U Diestelhorst J Germeshausen M Brandes G Lee-Gossler J Noyan F A Syndrome with Congenital Neutropenia and Mutations in G6PC3 N Engl J Med 2009 360 32 43 10.1056/NEJMoa0805051 19118303 PMC2778311

[B10] Banka S Chervinsky E Newman WG Crow YJ Yeganeh S Yacobovich J Donnai D Shalev S Further delineation of the phenotype of severe congenital neutropenia type 4 due to mutations in G6PC3 Eur J Hum Genet 2011 19 18 22 10.1038/ejhg.2010.136 20717171 PMC3039503

[B11] Boztug K Rosenberg PS Dorda M Banka S Moulton T Curtin J Rezaei N Corns J Innis JW Avci Z Tran HC Pellier I Pierani P Fruge R Parvaneh N Mamishi S Mody R Darbyshire P Motwani J Murray J Buchanan GR Newman WG Alter BP Boxer LA Donadieu J Welte K Klein C Extended spectrum of human glucose-6-phosphatase catalytic subunit 3 deficiency: novel genotypes and phenotypic variability in severe congenital neutropenia J Pediatr 2012 160 679 683 10.1016/j.jpeds.2011.09.019 22050868

[B12] Banka S Newman WG Özgül RK Dursun A Mutations in the G6PC3 gene cause Dursun syndrome Am J Med Genet 2010 152A 2609 2611 10.1002/ajmg.a.33615 20799326

[B13] Banka S Wynn R Byers H Arkwright PD Newman WG G6PC3 mutations cause non-syndromic severe congenital neutropenia Mol Genet Metab 2013 108 138 141 10.1016/j.ymgme.2012.12.001 23298686

[B14] Smith BN Evans C Ali A Ancliff PJ Hayee B Segal AW Hall G Kaya Z Shakoori AR Linch DC Gale RE Phenotypic heterogeneity and evidence of a founder effect associated with G6PC3 mutations in patients with severe congenital neutropenia Br J Haematol 2012 158 146 149 10.1111/j.1365-2141.2012.09110.x 22469094 PMC4533883

[B15] Donadieu J Fenneteau O Beaupain B Mahlaoui N Bellanne Chantelot C Congenital neutropenia: diagnosis, molecular bases and patient management Orphanet J Rare Dis 2011 6 26 10.1186/1750-1172-6-26 21595885 PMC3127744

[B16] Rezaei N Moazzami K Aghamohammadi A Klein C Neutropenia and Primary Immunodeficiency Diseases Int Rev Immunol 2009 28 335 366 10.1080/08830180902995645 19811314

[B17] Xia J Bolyard AA Rodger E Stein S Aprikyan AA Dale DC Link DC Prevalence of mutations in ELANE, GFI1, HAX1, SBDS, WAS and G6PC3 in patients with severe congenital neutropenia Br J Haematol 2009 147 535 542 10.1111/j.1365-2141.2009.07888.x 19775295 PMC2783282

[B18] Dursun A Ozgul RK Soydas A Tugrul T Gurgey A Celiker A Barst RJ Knowles JA Mahesh M Morse JH Familial pulmonary arterial hypertension, leucopenia, and atrial septal defect: a probable new familial syndrome with multisystem involvement Clin Dysmorphol 2009 18 19 23 10.1097/MCD.0b013e32831841f7 19011569

[B19] Bégin P Patey N Mueller P Rasquin A Sirard A Klein C Haddad E Drouin E Deist FL Inflammatory Bowel Disease and T cell Lymphopenia in G6PC3 Deficiency J Clin Immunol 2012 33 520 525 23180359 10.1007/s10875-012-9833-6

[B20] McDermott DH De Ravin SS Jun HS Liu Q Priel DAL Noel P Takemoto CM Ojode T Paul SM Dunsmore KP Hilligoss D Marquesen M Ulrick J Kuhns DB Chou JY Malech HL Murphy PM Severe congenital neutropenia resulting from G6PC3 deficiency with increased neutrophil CXCR4 expression and myelokathexis Blood 2010 116 2793 2802 10.1182/blood-2010-01-265942 20616219 PMC2974587

[B21] Banka S Wynn R Newman WG Variability of bone marrow morphology in G6PC3 mutations: Is there a genotype-phenotype correlation or age-dependent relationship? Am J Hematol 2011 86 235 237 21264919 10.1002/ajh.21930

[B22] Fernandez BA Green JS Bursey F Barrett B MacMillan A McColl S Fernandez S Rahman P Mahoney K Pereira SL Scherer SW Boycott KM Woods MO Adult siblings with homozygous G6PC3 mutations expand our understanding of the severe congenital neutropenia type 4 (SCN4) phenotype BMC Med Genet 2012 13 111 10.1186/1471-2350-13-111 23171239 PMC3523052

[B23] Cullinane AR Vilboux T O’Brien K Curry JA Maynard DM Carlson-Donohoe H Ciccone C Markello TC Gunay-Aygun M Huizing M Gahl WA Homozygosity mapping and whole-exome sequencing to detect SLC45A2 and G6PC3 mutations in a single patient with oculocutaneous albinism and neutropenia J Invest Dermatol 2011 131 2017 2025 10.1038/jid.2011.157 21677667 PMC3174312

[B24] Rubin GP Hungin APS Kelly PJ Ling J Inflammatory bowel disease: epidemiology and management in an English general practice population Aliment Pharmacol Ther 2000 14 1553 1559 10.1046/j.1365-2036.2000.00886.x 11121902

[B25] Humbert M Labrune P Simonneau G Severe pulmonary arterial hypertension in type 1 glycogen storage disease Eur J Pediatr 2002 161 S93 S96 12373580 10.1007/s00431-002-1012-y

[B26] Germeshausen M Zeidler C Stuhrmann M Lanciotti M Ballmaier M Welte K Digenic mutations in severe congenital neutropenia Haematologica 2010 95 1207 1210 10.3324/haematol.2009.017665 20220065 PMC2895047

[B27] Aytekin C Germeshausen M Tuygun N Dogu F Ikinciogullari A A Novel G6PC3 Gene Mutation in a Patient With Severe Congenital Neutropenia J Pediatr Hematol Oncol 2013 35 e81 e83 10.1097/MPH.0b013e3182679000 23018568

[B28] Gatti S Boztug K Pedini A Pasqualini C Albano V Klein C Pierani P A Case of syndromic neutropenia and mutation in G6PC3 J Pediatr Hematol Oncol 2011 33 138 10.1097/MPH.0b013e3181f46bf4 21285905

[B29] Jun HS Lee YM Song KD Mansfield BC Chou JY G-CSF improves murine G6PC3-deficient neutrophil function by modulating apoptosis and energy homeostasis Blood 2011 117 3881 3892 10.1182/blood-2010-08-302059 21292774 PMC3083300

[B30] Hayee BH Antonopoulos A Murphy EJ Rahman FZ Sewell G Smith BN McCartney S Furman M Hall G Bloom SL Haslam SM Morris HR Boztug K Klein C Winchester B Pick E Linch DC Gale RE Smith AM Dell A Segal AW G6PC3 mutations are associated with a major defect of glycosylation: a novel mechanism for neutrophil dysfunction Glycobiology 2011 21 914 924 10.1093/glycob/cwr023 21385794 PMC3110488

[B31] Jun HS Cheung YY Lee YM Mansfield BC Chou JY Glucose-6-phosphatase-β , implicated in a congenital neutropenia syndrome, is essential for macrophage energy homeostasis and functionality Blood 2012 119 4047 4055 10.1182/blood-2011-09-377820 22246029 PMC3350367

[B32] Ghosh A Shieh J-J Pan C-J Chou JY Histidine 167 is the phosphate acceptor in glucose-6-phosphatase-β forming a phosphohistidine enzyme intermediate during catalysis J Biol Chem 2004 279 12479 12483 14718531 10.1074/jbc.M313271200

[B33] Brunak S Engelbrecht J Knudsen S Prediction of human mRNA donor and acceptor sites from the DNA sequence J Mol Biol 1991 220 49 65 10.1016/0022-2836(91)90380-O 2067018

[B34] Sundström G Larsson TA Larhammar D Phylogenetic and chromosomal analyses of multiple gene families syntenic with vertebrate Hox clusters BMC Evol Biol 2008 8 254 10.1186/1471-2148-8-254 18803835 PMC2566581

[B35] Pan C-J Lei K-J Annabi B Hemrika W Chou JY Transmembrane topology of glucose-6-phosphatase J Biol Chem 1998 273 6144 6148 10.1074/jbc.273.11.6144 9497333

[B36] Chou JY Mansfield BC Mutations in the glucose-6-phosphatase-α (G6PC) gene that cause type Ia glycogen storage disease Hum Mutat 2008 29 921 930 10.1002/humu.20772 18449899 PMC2475600

[B37] Alizadeh Z Fazlollahi MR Eshghi P Hamidieh AA Ghadami M Pourpak Z Two cases of syndromic neutropenia with a report of novel mutation in G6PC3 Iran J Allergy Asthma Immunol 2011 10 227 230 21891829

[B38] Aróstegui JI De Toledo JS Pascal M García C Yagüe J Díaz de Heredia C A novel G6PC3 homozygous 1-bp deletion as a cause of severe congenital neutropenia Blood 2009 114 1718 1719 10.1182/blood-2009-04-219451 19696212

